# Complete Genome Sequences of Noncoding Regions of Korean Equine H3N8 Influenza Virus

**DOI:** 10.1128/genomeA.00461-14

**Published:** 2014-05-15

**Authors:** Woonsung Na, Minki Hong, Minju Yeom, Sanghyun Kim, Jeong-Ki Kim, Daesub Song

**Affiliations:** a Viral Infectious Disease Research Center, Korea Research Institute of Bioscience and Biotechnology, Daejeon, South Korea; b University of Science and Technology, Daejeon, South Korea; c College of Pharmacy, Korea University, Sejong, South Korea

## Abstract

We analyzed the complete genome sequence containing the 3′ and 5′ noncoding regions (NCRs) of the Korean H3N8 equine influenza virus (EIV), which will provide a better understanding of the pathogenesis, transmission, and evolution of EIV.

## GENOME ANNOUNCEMENT

Equine influenza virus (EIV), which is a member of the genus *Influenzavirus*, family *Orthomyxoviridae*, contains single-stranded negative-sense RNA of the following 8 gene segments: polymerase basic 2 (PB2), polymerase basic 1 (PB1), polymerase acidic (PA), hemagglutinin (HA), nucleoprotein (NP), neuraminidase (NA), matrix (M), and nonstructural (NS). EIV is classified according to the arrangement of two surface glycoproteins, HA and NA (1). Until now, two subtypes of EIV, H7N7 and H3N8, have been isolated from horses (2). Recently, Korean H3N8 EIV (strain A/equine/Kyonggi/SA1/2011) was isolated from a horse showing typical symptoms of respiratory disease, and the virus was found to belong to the Florida sublineage clade 1. It is unique that the virus possesses a naturally truncated NS gene segment and shows deduced viral replication in an *in vitro* kinetic study of growth kinetics (3), which is thought be affected by its NS truncation (4). To study other factors of replication, a sequence analysis of noncoding regions (NCRs) was required, as these regions have multiple functions in the replication of influenza viruses, such as polymerase binding, cap-snatching, transcription initiation, and packaging (5–8).

Viral RNA was extracted from the allantoic fluid of virus-inoculated embryonated chicken eggs using the RNeasy minikit (Qiagen, Inc., Valencia, CA), and the viral RNAs were circularized with T4 RNA ligase, as described previously (9–11). One-step reverse transcriptase PCR was employed to amplify each of the circularized viral gene segments using the OneStep RT-PCR kit (Qiagen, Inc.) and gene-specific primers. The amplified gene segments were purified with a QIAquick gel extraction kit (Qiagen, Inc.) and then sequenced bidirectionally using universal primer sets (12), and the sequences were confirmed 3 times at Cosmo Genetech, Ltd. (Seoul, Republic of Korea).

The complete genome of A/equine/Kyonggi/SA01/2011 (H3N8) is 13,596 nucleotides long; segments 1 to 8 are 2,341, 2,341, 2,233, 1,762, 1,565, 1,460, 1,027, and 867 nucleotides (nt), respectively, and its genes encode 11 proteins with amino acid lengths of 759 for PB2, 757 for PB1, 81 for PB1-F2, 716 for polymerase, 565 for HA, 498 for NP, 470 for NA, 252 for M1, 97 for M2, 121 for NS2 proteins, and a nonfunctional NS1 due to truncation. The virus has conserved 13 nucleotides (CCTTGTTTCTACT) at the 5′ NCR of 8 segments and 12 nucleotides at the 3′ NCR (AGCAAAAGCAGG), but PB2, PB1, and PA have a G at the 4th position of the 3′ NCR (AGCGAAAGCAGG).

Here, we describe the NCR sequences of A/equine/Kyonggi/SA1/2011(H3N8), and this is the first report of the full-genome sequence containing the 3′ and 5′ NCRs of H3N8 equine influenza virus. We hope this information will facilitate further investigations of the pathogenicity, transmission, and evolution of equine influenza viruses.

### Nucleotide sequence accession numbers.

The complete genome sequence of the A/equine/Kyonggi/SA01/Korea/2011 (H3N8) strain has been deposited and updated in Genbank under accession no. JX844143 to JX844150.
